# Mobile phones affect multiple sperm quality traits: a meta-analysis

**DOI:** 10.12688/f1000research.2-40.v1

**Published:** 2013-02-12

**Authors:** Madhukar Shivajirao Dama, M Narayana Bhat

**Affiliations:** 1 Institute of Wildlife Veterinary Research, Kodagu District, Karnataka, India

## Abstract

As mobile phone usage is growing rapidly, there is a need for a comprehensive analysis of the literature to inform scientific debates about the adverse effects of mobile phone radiation on sperm quality traits. Therefore, we conducted a meta-analysis of the eligible published research studies on human males of reproductive age. Eleven studies were eligible for this analysis. Based on the meta-analysis, mobile phone use was significantly associated with deterioration in semen quality (Hedges’s g = -0.547; 95% CI: -0.713, -0.382; p < 0.001). The traits particularly affected adversely were sperm concentration, sperm morphology, sperm motility, proportion of non-progressive motile sperm (%), proportion of slow progressive motile sperm (%), and sperm viability. Direct exposure of spermatozoa to mobile phone radiation with *in vitro* study designs also significantly deteriorated the sperm quality (Hedges’s g = -2.233; 95% CI: -2.758, -1.708; *p *< 0.001), by reducing straight line velocity, fast progressive motility, Hypo-osmotic swelling (HOS) test score, major axis (µm), minor axis (µm), total sperm motility, perimeter (µm), area (µm ^2^), average path velocity, curvilinear velocity, motile spermatozoa, and  acrosome reacted spermatozoa (%). The strength of evidence for the different outcomes varied from very low to very high. The analysis shows that mobile phone use is possibly associated with a number of deleterious effects on the spermatozoa.

## Introduction

Almost 10% of men of reproductive age are estimated to be subfertile ^1^. Owing to its complexity, even after identification of a plethora of underlying factors, etiology in almost half of the infertile subjects tested at fertility clinics remains obscure ^2^. Hence, the list of the causes of male infertility is growing by the day with recent advances in fertility research ^3^. Though advances in assisted reproduction technologies (ARTs), especially in the form of *in vitro* fertilization (IVF) and intracytoplasmic sperm injection (ICSI), have helped subfertile couples conceive offspring, it is feared that ARTs only bypasses the problem of subfertility and contributes towards hiding the underlying causes which have at times led to serious health problems in offspring ^4, 5^. Hence, identification of unknown aetiologies would help in prescription of specific preventive measures that will ultimately decrease the incidence of male infertility.

Most nations, especially developing countries, are witnessing an increase in the use of various radiation-emitting domestic-purpose devices that could cause mild to serious health problems based on the duration and intensity of usage ^6^, and reduced fertility is now recognised as one such problem ^7^. Wireless mobile phones are one of the most accepted devices with a tremendous increase in usage across the world in recent times ^8^. Research into the impact of ionizing radiation on the development of various types of health disorders, especially cancers, has been well established ^9^. Similarly, several studies have found an increase in the risk of developing some types of tumors after long-term exposure to non-ionizing radiation from mobile phones ^10^. Research into the effects of mobile phone radiation on male fertility, though growing, is limited and inconclusive ^11, 12^. Recently, several case-control studies have reported results from a general population setting alongside a few studies from subfertile populations ^7, 13– 20^. Like ionizing radiation, non-ionizing radiation is also expected to affect spermatozoa, though in subtle ways ^21^. The aim of this meta-analysis was, therefore, to investigate the impact of mobile phone radiation on semen parameters *in vitro* as well as *in vivo* settings in men of reproductive age from both general and subfertile populations.

## Material and methods

A systematic search of an electronic database was conducted to retrieve published studies on the impact of mobile phone radiation on semen parameters in adult men. The results have been reported according to the standards of the guidelines for meta-analysis of observational studies in epidemiology ^22^. All English language research studies published up until January 2012 in scientific journals indexed in the searched databases were included for analysis.

*Inclusion/exclusion criteria and outcomes of interest:* The studies on human males of reproductive age reporting the effect of mobile phone radiation on any or all measures of semen volume, total sperm count, sperm concentration, sperm motility or sperm morphology were included. All the studies that did not satisfy the inclusion criteria were excluded.

*Search strategy, data extraction and meta-analysis:* Google Scholar and NLM’s PubMed database were searched for articles by using different combinations of 4 mobile phone related keywords [‘mobile phone’, ‘cellular phone’, ‘radiofrequency electromagnetic waves (RF-EMW)’, ‘radiation’] with 5 sperm quality related keywords (‘spermatozoa’, ‘semen’, ‘sperm concentration’, ‘sperm motility’, ‘sperm morphology’) Data from 11 eligible studies were extracted and separated into *in vitro* and *in vivo* categories.

Effect sizes were expressed as Hedges’s g ^23^, separately for *in vivo* & *in vitro* studies using individual semen parameters as units of analysis ( Supplementary Table 1). A random model was used to test and quantify effect size using ‘Comprehensive Meta-Analysis (v.2)’ trial version ^24^. A random effect model was preferred over a fixed effect model in order to account for differences in both effect size and sampling error ^25^.

## Results

### *In vivo* effects of mobile phone radiation

Our analysis shows that overall, mobile phone users had significant deterioration in semen quality (Hedges’s g = -0.547; 95% CI: -0.713, -0.382; *p* < 0.001). There was significant heterogeneity among effect sizes (Q = 475.985, p < 0.001), which suggest that some of the semen parameters may not be affected by mobile phone exposure. Hence, combined effect-size for each of the semen parameters were calculated separately ( Table 1), and it was found that sperm concentration, sperm morphology, sperm motility, proportion of non-progressive motile sperm (%), proportion of slow progressive motile sperm (%), and sperm viability were deteriorated in individuals exposed to mobile phone radiation. By contrast, semen volume, liquefaction time, semen pH, proportion of rapid progressive motile sperm (%), and semen viscosity were not affected by mobile phone usage.

**Table 1.** Effect sizes of mobile phone radiation on sperm quality traits.

	Sample size	Hedges’s g	*p*-value
*In vivo* studies			
Semen volume	591	0.09774	0.29458
Sperm concentration	874	-0.66388	0.01858
Sperm morphology	746	-1.28325	0.00000
Sperm motility	1079	-0.81584	0.00102
Proportion of non-progressive motile sperm (%)	283	-0.16136	0.03396
Proportion of rapid progressive motile sperm (%)	283	-0.25708	0.09969
Proportion of slow progressive motile sperm (%)	283	-0.39031	0.00765
Liquefaction time (min)	321	-0.11449	0.28277
pH	321	-0.36681	0.05592
Sperm viability (%)	321	-1.13150	0.00220
Semen viscosity	321	-0.00924	0.93083
*In vitro* studies			
Acrosome reaction (%)	24	-1.69939	0.00000
Sperm area (µm ^2^)	24	-6.79952	0.00004
Average path velocity	20	-8.16777	0.00000
Curvilinear velocity	20	-10.37987	0.00000
DNA fragmentation	32	0.10182	0.68034
Fast progressive motility	49	-0.50794	0.01195
Hypo-osmotic swelling (HOS)	20	1.721867	0.000002
Major axis (µm)	24	-3.62708	0.01918
Minor axis (µm)	24	-7.4825	0.0361
Sperm motility	105	-2.82739	0.00118
Non motile spermatozoa	49	-0.61615	0.03275
Non progressive motility	49	0.04371	0.82612
Perimeter (µm)	24	-5.53132	0.01897
Progressive motility	12	-0.04606	0.90700
Reactive oxygen species (ROS)	36	-11.37087	0.33592
Slow progressive motility	49	-0.14543	0.67535
Sperm concentration	59	-0.02309	0.89887
Sperm zona binding	10	-0.68402	0.12153
Straight line velocity	20	-6.37614	0.00000
Total antioxidant capacity (TAC)	32	-0.25102	0.31138
Viability (%)	56	-2.75116	0.02543

Publication bias could potentially change the results of meta-analysis but analysis of funnel plot of precision by Hedges’s g using Dual and Tweedie’s trim-and-fill test ^26^ did not change the overall effect size, suggesting little bias. Moreover, Rosenthal’s fail-safe N test ^27^ revealed that 3964 missing studies with a mean Hedges’s g of 0 are required for the combined 2-tailed p-value to exceed 0.050. In other words, there need to be 99.1 missing studies for every observed study for the effect to be nullified.

### *In vitro* effects of mobile phone radiation

Experimental exposure of spermatozoa isolated from healthy men of reproductive age to mobile phone radiation significantly affected sperm quality (Hedges’s g = -2.233; 95% CI: -2.758, -1.708; *p* < 0.001). There was significant heterogeneity among effect sizes (Q = 639.294, p<0.001), suggesting that similar to *in vivo* exposure, *in vitro* exposure may also not affect all the parameters of spermatozoa. Hence, combined effect-size for spermatozoa parameters were calculated separately ( Table 1), and it was found that exposure to mobile phones significantly reduced straight line velocity, fast progressive motility, Hypo-osmotic swelling (HOS) test score, major axis (µm), minor axis (µm), total sperm motility, perimeter (µm), area (µm ^2^), average path velocity, curvilinear velocity, motile spermatozoa, and acrosome reacted spermatozoa (%). By contrast, DNA fragmentation levels, non-progressive motility, total antioxidant capacity (TAC), progressive motility, reactive oxygen species (ROS) generation, slow progressive motility, sperm concentration, and sperm zona binding was not affected by mobile phone radiation.

A Funnel plot of precision by Hedges’s g using Dual and Tweedie’s trim-and-fill test did not change the overall effect size, suggesting little publication bias. Rosenthal’s fail-safe N test revealed that 3813 missing studies with a mean Hedges’s g of 0 are required for the combined 2-tailed p-value to exceed 0.050. In other words, there need to be 100.3 missing studies for every observed study for the effect to be nullified.

## Discussion

This study was aimed to analyse the data assessing the risk of mobile phone radiation on male fertility. Our results suggest that mobile phone radiation has a tendency to significantly affect sperm quality. Based on the design of the analysed records, we divided studies into *in vivo* studies and *in vitro* studies. The effect size was significant in both the categories, suggesting that mobile phone radiation could severely compromise male fertility. This conclusion is robust, as a fail-safe test suggested that the results are not likely to be mediated by publication bias.

The number of worldwide mobile subscriptions grew from less than 1 billion in 2000 to over 6 billion in 2012 ^8^, with more than half of these subscribers estimated to be children and young adults. Hence, it is very likely that in the coming decades, we could witness an increase in the incidence of male infertility due to mobile phone radiation exposure, similar to growing concerns over other hazards. Although the mechanism of cell phone radiation-mediated health defects is still obscure, it is proposed that their ability to produce heat, disrupt cell membranes, affect endothelial function, alter the blood-brain barrier, and modulate neuronal excitability have the potential to affect multiple physiological functions simultaneously ^28– 30^.

To our knowledge, this is the first meta-analysis of the effects of mobile phone radiations on various sperm quality parameters. Cellular phones have become integral part of everyday life, and newer versions of these are developed very rapidly these days. Hence, it is necessary to educate the users about the hazards of cell phones as well as test the newer versions like smartphones for health hazards.

## Figures and Tables

**Supplementary Table 1.** Effect sizes of sperm quality traits from the studies included in the analysis.

Reference	Subgroup	Outcome	Effect size (Hedges’s g)	*p*-value
*In vivo* studies				
[ 7]	1	Proportion of non-progressive motile sperm (%)	-0.11444	0.19545
		Proportion of rapid progressive motile sperm (%)	-0.39434	0.00001
		Proportion of slow progressive motile sperm (%)	-0.51671	0.00000
		Sperm concentration	0.01922	0.82778
		Sperm motility	-0.14692	0.09667
	2	Proportion of non-progressive motile sperm (%)	-0.28478	0.04855
		Proportion of rapid progressive motile sperm (%)	-0.07945	0.58037
		Proportion of slow progressive motile sperm (%)	-0.22090	0.12525
		Sperm concentration	-0.12467	0.38594
		Sperm motility	0.00940	0.94784
[ 13]	1	Sperm morphology	-0.74105	0.00000
		Sperm motility	-0.57347	0.00000
[ 14]	1	Liquefaction time (min)	-0.01209	0.94773
		pH	0.00000	1.00000
		Semen volume	0.18269	0.32253
		Sperm concentration	-0.42958	0.02095
		Sperm morphology	-0.72462	0.00013
		Sperm motility	-0.40596	0.02896
		Viability	-0.43282	0.02002
		Viscosity	0.01942	0.91612
	2	Liquefaction time (min)	-0.23709	0.20389
		pH	-0.46407	0.01363
		Semen volume	-0.02014	0.91380
		Sperm concentration	-0.56141	0.00298
		Sperm morphology	-1.70950	0.00000
		Sperm motility	-1.32047	0.00000
		Viability	-1.34677	0.00000
		Viscosity	-0.09456	0.61148
	3	Liquefaction time (min)	-0.09749	0.59412
		pH	-0.63951	0.00060
		Semen volume	0.28711	0.11786
		Sperm concentration	-0.87694	0.00000
		Sperm morphology	-1.95983	0.00000
		Sperm motility	-1.58904	0.00000
		Viability	-1.62719	0.00000
		Viscosity	0.04490	0.80606
[ 15]	1	Semen volume	-0.07567	0.69348
		Sperm concentration	-2.09426	0.00000
		Sperm morphology	-1.35171	0.00000
		Sperm motility	-1.80265	0.00000
Overall effect			-0.54948	0.00000
*In vivo* studies				
[ 16]	1	Fast progressive motility	-0.48612	0.07419
		Motility	-0.73467	0.00808
		Non motile	-0.89668	0.00146
		Non progressive motility	0.14043	0.60105
		Slow progressive motility	-0.48268	0.07620
		Sperm concentration	-0.05135	0.84822
[ 20]	1	Dna fragmentation	0.10182	0.68034
		Motility	-0.19307	0.43544
		ROS	-0.29465	0.23542
		Sperm concentration	0.00085	0.99725
		TAC	-0.25102	0.31138
		Viability (%)	-0.46743	0.06193
[ 17]	1	Progressive motility	-0.04606	0.90700
[ 31]	1	Motility	-16.10595	0.00008
		ROS	-23.97770	0.00007
		Viability (%)	-11.52174	0.00009
[ 32]	1	Acrosome (%)	-1.58348	0.00051
		Area (µm ^2^)	-8.61098	0.00000
		Major axis (µm)	-5.25493	0.00000
		Minor axis (µm)	-11.21546	0.00000
		Perimeter (µm)	-8.00952	0.00000
		Sperm zona binding	-0.68402	0.12153
	2	Acrosome (%)	-1.82487	0.00012
		Area (µm ^2^)	-5.27741	0.00000
		Major axis (µm)	-2.15357	0.00002
		Minor axis (µm)	-4.06799	0.00000
		Perimeter (µm)	-3.28849	0.00000
[ 18]	1	Fast progressive motility	-0.53471	0.07618
		Motility	-0.64188	0.03467
		Non motile	-0.31928	0.28406
		Non progressive motility	-0.07395	0.80286
		Slow progressive motility	0.21209	0.47510
[ 19]	1	Average path velocity	-8.16777	0.00000
		Curvilinear velocity	-10.37987	0.00000
		HOS	1.72187	0.00000
		Motility	-9.78102	0.00000
		Straight line velocity	-6.37614	0.00000
		Viability (%)	-2.53934	0.00000
Overall effect size			-2.23292	0.00000
