# Supplementary material for: Mobile phones affect multiple sperm quality traits: a meta-analysis
Source: F1000Res. 2013 Feb 12;2:40. [Version 1] doi: 10.12688/f1000research.2-40.v1 (PMC3752730; doi:10.12688/f1000research.2-40.v1)
Supplement: Peer review history

# Peer review: Referee response for version 1

**Published:** 2013-03-25

**Authors:** Essam- Eldeen M Mohamed

**Affiliations:** Department of Dermatology and Andrology, Al-Azhar University Hospital, Assiut, Egypt

I have read this submission. I believe that I have an appropriate level of expertise to confirm that it is of an acceptable scientific standard.

# Peer review: Referee response for version 1

**Published:** 2013-03-14

**Authors:** Gary Klinefelter

**Affiliations:** Gamete and Early Embryo Biology Branch, Reproductive Toxicology Division, United States Environmental Protection Agency, Research Triangle Park, NC, USA

On the surface, the results seem quite striking with virtually any sperm endpoint one can imagine being significantly altered in the collective analysis of mobile phone studies compiled. However, upon looking at the data in the supplementary table, it is obvious that the relatively few studies compiled varied widely both in respect to endpoints measured and the sample size.  As shown in Table 1, motility is the endpoint representing the greatest combined sample size for both *in vivo* and *in vitro* studies. Motility was measured in 4 out of 4 *in vivo* studies and 5 out of 7 *in vitro* studies. So motility ‘might’ be an endpoint that is repeatedly altered by cell phone exposure.  The reason for ‘might’ is the lack of any reported exposure data in this study. 

In summary, the small sample size and lack of exposure data significantly weaken the conclusions of this study.

I have read this submission. I believe that I have an appropriate level of expertise to confirm that it is of an acceptable scientific standard, however I have significant reservations, as outlined above.

# Response

**Published:** 2013-04-02

**Authors:** Madhukar Dama

**Affiliations:** IWVR, KVAFSU, India

We have studied the reviewer comments and would like to justify our results. Our analysis is showing that mobile phone radiations could affect many sperm parameters. This could be due to interdependence of sperm parameters ( Acta Eur Fertil. 1982;13(2):49-54). We also agree with the point that the number of studies is few and total sample size in *in vitro* studies is smaller. However, it must be noted that the sample size is weighted during meta-analysis, which nullifies the problems posed by smaller sample size studies. Apart from motility, other parameters like morphology, concentration, and viability are also significantly affected by *in vivo* exposure. Hence we have provided all the effect sizes individually along with p values and sample size. We hope that our points justify the reviewer comments.

# Peer review: Referee response for version 1

**Published:** 2013-02-19

**Authors:** Nelson Bennett

**Affiliations:** Institute of Urology, Lahey Clinic Medical Center, Burlington, MA, USA

I have read this submission. I believe that I have an appropriate level of expertise to confirm that it is of an acceptable scientific standard.
